# The Normal Transverse Mesocolon and Involvement of the Mesocolon in Acute Pancreatitis: An MRI Study

**DOI:** 10.1371/journal.pone.0093687

**Published:** 2014-04-04

**Authors:** Xiao Xiao Chi, Xiao Ming Zhang, Tian Wu Chen, Xiao Hua Huang, Lin Yang, Wei Tang, Bo Xiao

**Affiliations:** Sichuan Key Laboratory of Medical Imaging, Department of Radiology, Affiliated Hospital of North Sichuan Medical College, Nanchong, Sichuan, PR China; Wayne State University, United States of America

## Abstract

**Objective:**

To study the MRI findings of the normal transverse mesocolon and the involvement of the mesocolon in acute pancreatitis (AP) as well as the relationship between the involvement of the mesocolon and the severity of AP.

**Materials and Methods:**

Forty patients without pancreatic disorders were retrospectively analyzed to observe the normal transverse mesocolon using MRI; 210 patients with AP confirmed by clinical and laboratory tests were retrospectively analyzed using MRI to observe transverse-mesocolon involvement (TMI). The severity of TMI was recorded as zero points (no abnormalities and transverse-mesocolon vessel involvement), one point (linear and patchy signal in the transverse mesocolon) or two points (transverse-mesocolon effusion). The AP severity was graded by the MRI severity index (MRSI) and the Acute Physiology And Chronic Healthy Evaluation II (APACHE II) scoring system. The correlations of TMI with MRSI and APACHE-II were analyzed.

**Results:**

In a normal transverse mesocolon, the display rates of the middle colic artery, the middle colic vein and the gastrocolic trunk on MRI were 95.0%, 82.5% and 100.0%, respectively. Of the 210 patients with AP, 130 patients (61.9%) had TMI. According to the TMI grading, 40%, 39% and 20% of the patients were graded at zero, one and two points, respectively. TMI was strongly correlated with the MRSI score (r = 0.759, P = 0.000) and the APACHE-II score (r = 0.384, P = 0.000).

**Conclusion:**

MRI could be used to visualize transverse-mesocolon involvement. The severity of TMI could reflect that of AP in the clinical setting and imaging. TMI might be a supplementary indicator of the severity of AP.

## Introduction

Acute pancreatitis (AP) is a common cause of acute-abdomen presentation in clinical practice. Acute pancreatitis leads to a wide range of local and systemic pathophysiological alterations and to a large variability in clinical manifestations and prognosis [1], [2]. Mild acute pancreatitis (MAP) has a mortality rate of <1% [3]; severe acute pancreatitis (SAP) has a mortality rate of 10–30% [4], [5], [6]. Assessment of the severity is crucial for the proper management of patients with AP.

AP is a process of inflammation diffusion [6], [7], and the diffusion could have a very wide range; it is important to respond to the clinical severity [8]. AP and intra-abdominal tumors could affect the omentum and mesentery [9]. Mesentery involvement indicates that the inflammation has spread from the retroperitoneal space to the subperitoneal space. Most researchers have focused on the AP involvement in the retroperitoneal space. The subperitoneal space involvement is as important to the response to the severity of AP. In this study, we aim to observe the MRI findings of AP involving the transverse mesocolon (served as one part of the subperitoneal space).

The ease, speed and availability of CT are important in the diagnosis of AP. Enhanced CT increases the rate of detection of AP; however, injury from CT radiation and the potential iodine toxicity to the pancreas and kidneys are major limitations [10]. CT is not sensitive to AP characterized by interstitial edema and mild acute pancreatitis (graded as A, B and C by the CTSI) [11], [12]. MRI could be used to assess inflammatory changes accurately, and the MR severity index (MRSI) could be used to determine the prognosis [13]; it clearly shows the areas of necrosis with no need for enhancement [14]. MRI is widely used in the diagnosis of AP and might be equivalent to CT in the detection of bowel-wall thickening and changes in the mesentery [15], or it be might superior to CT for observation, including observation of the peritoneum and mesentery [16].

We conducted this study to investigate retrospectively the normal findings of the transverse mesocolon in the control group and its involvement in acute pancreatitis using MRI. We hypothesized the following: (1) MRI could be used to visualize the normal transverse mesocolon; (2) MRI could be used to visualize the patterns of transverse-mesocolon involvement in acute pancreatitis; and (3) the severity of transverse-mesocolon involvement shown by MRI is correlated with that of acute pancreatitis graded by the MRSI and the Acute Physiology And Chronic Healthy Evaluation II (APACHE II) scores.

## Materials and Methods

### 1. Ethics Statement

The study was approved by the Institutional Review Board and the Ethics Committee of the Affiliated Hospital of North Sichuan Medical College. It is difficult to obtain informed consent from all of the patients involved in the study because of the retrospective nature of this study and the health care conditions in Nanchong. The Ethics Committee considered that the experimental design and the program of the study would not cause harm or risk to the subjects. The data collected were analyzed anonymously. The Ethics Committee of our Hospital waived the need for written informed consent from the participants. The study complies with the ethical principles of the Helsinki Declaration of 1964, as revised by the World Medical Organization in Edinburgh in 2000.

### 2. Patient Selection

The patients with AP admitted to our institution between April 2010 and March 2012 were considered for inclusion in this study. The diagnosis of acute pancreatitis was based on the presence of typical abdominal pain combined with three-fold elevated amylase or lipase. All of the readers were blinded to the clinical presentation, blood work and outcomes of the patients. The inclusion criteria for the patients in this study were as follows: (a) in-patient; (b) acute onset of abdominal pain; (c) pancreatitis at first onset; (d) three-fold elevated amylase or lipase, excluding other causes of elevated enzymes; and (d) abdominal MRI examination. The exclusion criteria in this study were as follows: (a) resistance to MR imaging (including contraindications to MRI, claustrophobia, inability to control breathing); (b) a history of chronic pancreatitis; (c) AP resulting from pancreatic carcinoma; and (d) the presence of other diseases with mesenteric involvement (including hypoproteinemi, chronic liver disease, peritoneal dialysis). A total of 210 patients with AP were enrolled in this study. All of the patients had a clinical assessment and laboratory workup on admission.

In the same period, 40 continuous patients with no mesentery disorders visible on MRI between September 2011 and March 2012 served as the control group. The subjects had no abdominal symptoms and signs, and on MRI, we found nothing that could cause mesentery involvement. An upper-abdominal examination (plain-scan and dynamic-enhanced) was performed in our hospital as part of a physical examination or to test for other diseases, and the results included 19 patients with no abnormal findings in the abdomen, eight patients with hepatic cysts, five patients with hepatic hemangioma and eight patients with renal cysts visualized on MRI.

### 3. MR-imaging Technique

All of the MR examinations were performed during suspended respiration with a 1.5-T system and a phased-array coil (Signa, GE Medical Systems, Milwaukee, WI, USA). The sequences included two-dimensional coronal and axial single-shot fast spin-echo (SSFSE) T2-weighted, axial fast-recovery fast-spin-echo (FRFSE) T2-weighted with fat suppressed, axial spoiled dual gradient-echo (GRE T1-weighted in- and out-of-phase, axial-slab three-dimensional (3D) spoiled gradient-echo (SPGR) dynamic contrast-enhanced with fat-suppressed and SSFSE radial series slab MRCP. The coronal SSFSE T2-weighted MR images were obtained in two or more breath-holds. The following parameters were used: repetition time [TR] ms/echo time [TE] ms, 2500∼3500/80–100; section thickness, 7 mm; intersection gap, 0; matrix, 256×192; signal acquired, one half; and field of view (FOV), 32×32 cm. The axial SSFSE T2-weighted images were obtained in one or two breath-holds. The following parameters were used: TR ms/TE ms, 2500∼3500/80–100; section thickness, 5 mm; intersection gap, 0; matrix, 256×160; signal acquired, one half; and FOV, 32×24 cm. The axial two-dimensional multi-section SPGR T1-weighted images were obtained during breath holding. The following parameters were used: TR ms/TE ms, 120/4.2 (in-phase) and 2.1 (out-of-phase); flip angle, 90°; section thickness, 8 mm; intersection gap, 0; matrix, 256×192; signal acquired, 1; and field of view, 32×24 cm. SSFSE radial oblique slabs were obtained for MR cholangiopancreatography (MRCP) with the following parameters: TR ms/TE ms 6000/700, fat saturation, 40-mm section thickness, 256×192 matrix, half signal acquired, and 24×24 cm FOV. The axial three-dimensional SPGR dynamic MR images were obtained with TR ms/TE ms, 6.1/2.1; flip angle, 15–20°; matrix, 256 ×128; signal acquired, 1; section thickness, 5 mm; overlap, 2.5 mm; and FOV, 32×24 cm. Three-dimensional SPGR was obtained at 2.5 mm increments with zero-fill interpolation for dynamic enhancement. Twenty milliliters of gadolinium (Magnevist; Schering Guangzhou Co., China) was administered intravenously with a pressure injector (Spectris MR Injection System, Medrad, Inc., USA) at 2–3 ml/s and followed by a 20-ml saline solution flush. The first-pass arterial enhancement was optimized with a timing bolus sequence (axial FMPSPGR). The dynamic imaging was performed during breath holding before the injection (unenhanced), immediately after the injection (hepatic arterial phase), 30 s after the injection (early venous phase) and 1 min after the injection (late venous phase). The delayed phases (90 s after the injection) were acquired with axial fast spoiled gradient echo (FSPGR) and 3D SPGR T1-weighted sequences.

### 4. MRI Images Review

The original MRI data were loaded to a workstation (GE, AW 4.4) for observation and measurement. The MR images were reviewed by two observers (with more than three years of experience in abdominal MR imaging), who were blinded to the laboratory data and clinical outcomes.

#### 4.1. Normal transverse mesocolon on MRI

The features of the normal transverse mesocolon observed on MRI included the transverse mesocolon adipose tissue signal (uniformity, consistency with the subcutaneous fat signal on the T2-weighted image in the axial and coronal views); the shape and direction of the mesenteric vessels, with a focus on the middle colic artery, middle colic vein and gastrocolic trunk in the axial and coronal views; the diameter of the above three vessels on dynamic enhanced MRI; the lymph nodes attached to the transverse mesocolon; and the size of the root of the transverse mesocolon (if larger than 5 mm in the short diameter).

#### 4.2. MRI findings in acute pancreatitis on MRI

AP was defined as edematous and necrotic pancreatitis on the MR images [17], [18], [19]. The severity of AP on MRI was graded according to the MR-severity index (MRSI), which was derived from the CT-severity index [20]. AP was graded as mild (0–3 points), moderate (4–6 points) or severe (7–10 points), according to the MRSI [20], [21], [22].

#### 4.3. Transverse-mesocolon involvement on MRI

The findings of transverse-mesocolon involvement observed on MRI included transverse-mesocolon edema, thickening, effusion and transverse-mesocolon vessel involvement. We measured the diameter of the middle colic artery, middle colic vein and gastrocolic trunk in the patients with AP who had received dynamic enhanced MR imaging. The severity of TMI was graded as Grade I (included no abnormalities and transverse-mesocolon vessels involvement, zero points) Grade II (linear and patchy signal in the transverse mesocolon, one point); or Grade III (transverse-mesocolon effusion, two points). The transverse mesocolon is divided into right and left by the centerline of the body.

### 5. The APACHE-II Score

The APACHE II is widely used to evaluate the severity of acute pancreatitis in clinical practice [23]. An APACHE-II score of eight is a cut-off point to differentiate between mild AP (zero to seven points) and severe AP (≥eight points) [4]. Two physicians with five years of experience in treating digestive diseases calculated the scores according to the laboratory data and clinical outcomes.

### 6. Statistical Analysis

The MRSI and TMI scores on the MRI were averaged between the two observers. Any discrepancies between the readers were settled by consensus. Kappa statistics were used to assess the inter-rater reliability.

The continuous variables were expressed as the mean ± SD and the range, and the T-test was used to analyze the difference between the AP group and the control group in the blood vessels of the transverse mesocolon and patient age. The matched four-fold table chi-square test was used to analyze the difference in the sex ratio between the AP group and the control group. Chi-squared tests were used to analyze the differences in the transverse-mesocolon involvement between mild, moderate and severe AP according to the MRSI and between mild AP and severe AP according to the APACHE-II scoring system. The Spearman rank-correlation coefficient was calculated to test the correlation of the transverse-mesocolon involvement score with the MRSI and the APACHE-II score.

The statistical tests were performed using the Statistical Package for Social Sciences (SPSS) for Windows (Version 13.0, Chicago, IL, USA). P-values <0.05 were considered significant.

## Results

### 1. Patient Sample

#### 1.1. Control group

The 40 patients serving as the controls included 24 males and 16 females, with a mean age of 49±10 years (range 26–70 years). All of the patients had received a plain scan and dynamic enhanced MR imaging.

#### 1.2. Acute pancreatitis

The 210 patients with AP included 107 women and 103 men with a mean age of 51±15 years (range 17–87 years). The difference was not statistically significant in age (T = 1.095, P = 0.277) and the sex ratio (χ2 = 1.613, P = 0.204) between the acute pancreatitis group and the control group. We hypothesized that the groups did not have remarkable differences in age and sex ratio.

Of the patients, 76 (36.2%) had received plain scan and dynamic enhanced MR imaging, whereas 134 (63.8%) had received plain scan.

In the 210 cases with AP, the etiology of AP was biliary in 117 (55.7%) cases, hyperlipidemia-related in 34 (16.2%) cases, alcohol-related in 15 (7.1%) cases, pregnancy-related in five (2.4%) cases, surgery-related in three (1.4%) cases and unknown in 36 (17.1%) cases.

### 2. Findings of Normal Transverse Mesocolon on MRI

The agreement between the observers for the findings of normal transverse mesocolon was good (κ = 0.821, *P*<0.001).

**Figure 1:**
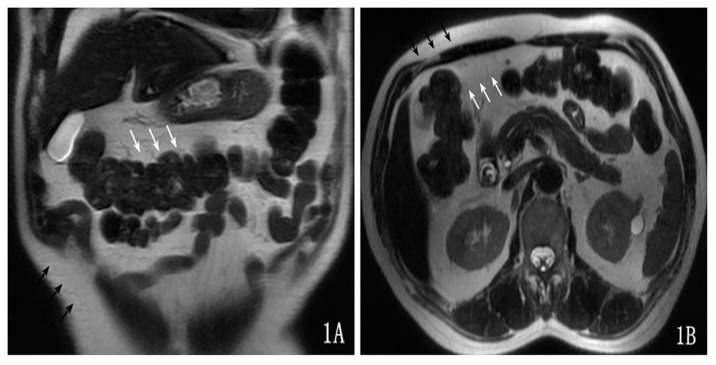
The signal intensity of the transverse-mesocolon adipose tissue was uniform (white arrow) and consistent with the subcutaneous fat (black arrow) signal on the T2-weighted image.

The transverse mesocolon adipose tissue signal was uniform, and it was consistent with the subcutaneous fat signal on the T2-weighted image (figure 1).

**Figure 2:**
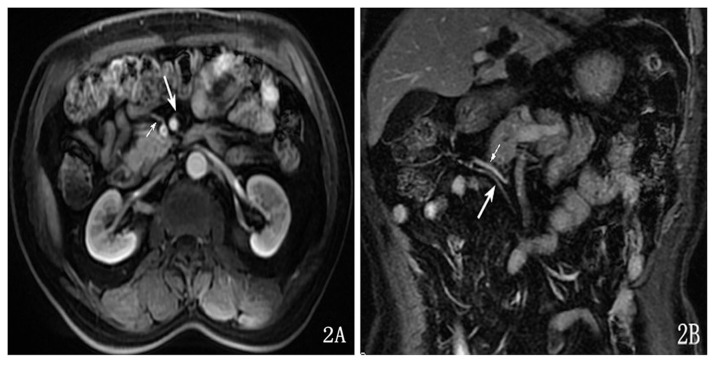
The middle colic artery showed on the axial enhancement image (A, long arrow) and on the coronal enhancement image (B, long arrow). The middle colic vein was shown on the axial enhancement image (A, short arrow) and on the coronal enhancement image (B, short arrow).

The display rate of the middle colic artery was 95.0% (38/40). Among these cases, the display rate was 95.0% (38/40) in the axial view and 85.0% (34/40) in the coronal view. The display rate of the combination of axial and coronal planes is higher than that of the coronal planes; however, the difference was not statistically significant (P>0.05). The middle colic artery can be visualized in the arterial phase, and 95.0% of the middle colic arteries originated at the anterior or right anterior wall of the superior mesenteric artery (SMA) and progressed to the upper right (figure 2); 5.0% of the cases originated at the left anterior wall and the right posterior wall. The diameter of the trunk of the middle colic artery was 1.52±0.23 mm.

The display rate of the middle colic vein was 82.5% (33/40). Among these cases, the display rate was 80.0% (32/40) in the axial view and 67.5% (27/40) in the coronal view. The display rate of the combination of the axial and coronal planes is higher higher than that of the coronal planes (P<0.05). This vein could be observed in the early and late venous phases. The vein was concomitant with the middle colic artery. Its abouchement was the anterior or right anterior wall of the superior mesenteric vein (SMV); in 92.5% of the cases, the vein progressed to the lower right (figure 2), and in 7.5% of the cases, it progressed to the upper right. The diameter of the trunk of the vein was approximately 2.19±0.23 mm.

**Figure 3:**
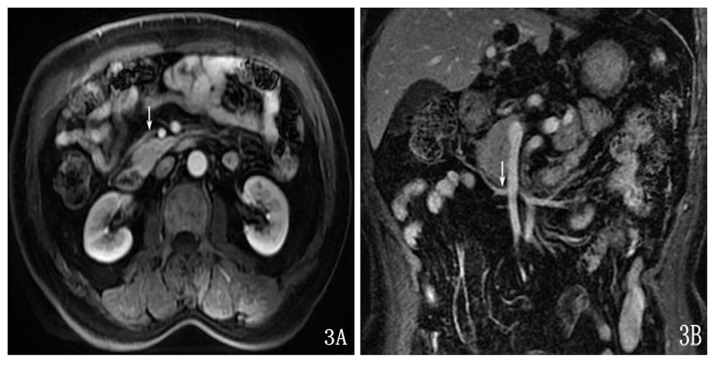
The gastrocolic trunk was shown on the axial enhancement image (A) and on the enhancement coronal image (B).

The display rate of the gastrocolic trunk was 100.0% (40/40) in the axial and coronal view. It could be visualized in the early and late venous phases. The abouchement of the gastrocolic trunk was the right wall of the SMV, and the vein progressed transversely (figure 3). The diameter of the gastrocolic trunk was 4.36±0.55 mm.

**Figure 4:**
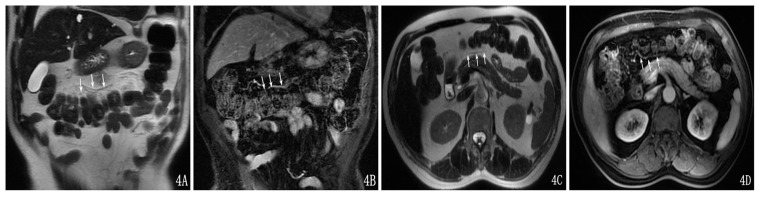
The transverse-mesocolon vessels were shown on the coronal SSFSE T2-weighted image (A), enhancement on the coronal image (B), on the axial SSFSE T2-weighted image (C) and enhancement on the axial image (D).

The transverse mesocolon vessels demonstrated hypointensity on T2WI compared with fat (figure 4) and were markedly enhanced after the enhanced scan.

There was no lymph node larger than 5 mm at the transverse mesocolon; in 5.0% of the patients (2/40), a lymph node was displayed at the root of the transverse mesocolon. Both patients had a single lymph node, less than 5 mm in diameter.

### 3. Findings of Acute Pancreatitis on MRI

The agreement between the observers for the findings of AP on MRI was good (κ = 0.801, P<0.001).

Of the 210 patients with AP, 148 patients (70%) were diagnosed with edematous AP, whereas 62 patients (30%) were diagnosed with necrotizing AP on MRI. The mean MRSI score was 4.13±1.79 (ranging from one to 10). Among the 62 patients with necrotizing AP, 44 had necrosis of less than 30% of the total pancreatic volume, 17 had necrosis of 30 to 50%, and one had necrosis of more than 50%. Additionally, 37.1%, 54.8% and 8.1% had mild, moderate and severe AP, respectively, according to the MRSI.

### 4. Findings of Transverse-mesocolon Involvement on MRI

**Figure 5:**
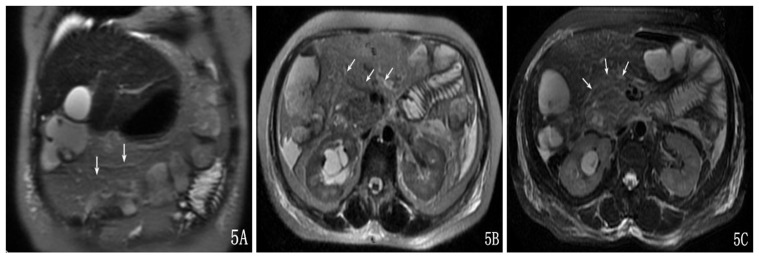
A 56-year-old female with moderate AP and TMI was Grade II. The transverse mesocolon showed edema and thickening on a coronal SSFSE T2-weighted image (A), on an axial SSFSE T2-weighted image (B) and on an axial SSFSE T2-weighted image with fat suppression (C).

**Figure 6:**
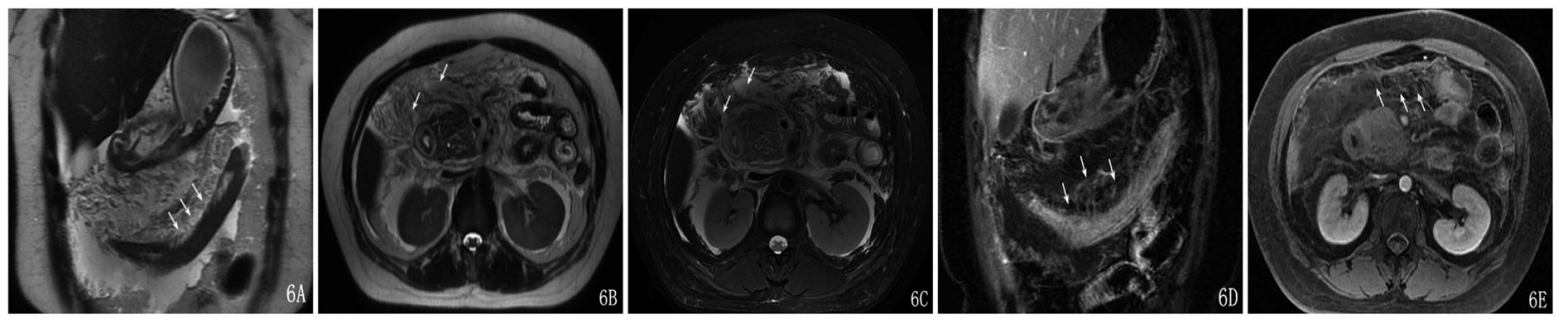
A 47-year-old female with severe AP and TMI was Grade III. The transverse mesocolon showed edema and thickening on a coronal SSFSE T2-weighted image (A). The transverse mesocolon showed effusions on an axial SSFSE T2-weighted image (B) and on an axial SSFSE T2-weighted image with fat suppression (C). The transverse-mesocolon-vessel involvement could be seen on the enhancement of the coronal image (D) and axial image (E).

**Figure 7:**
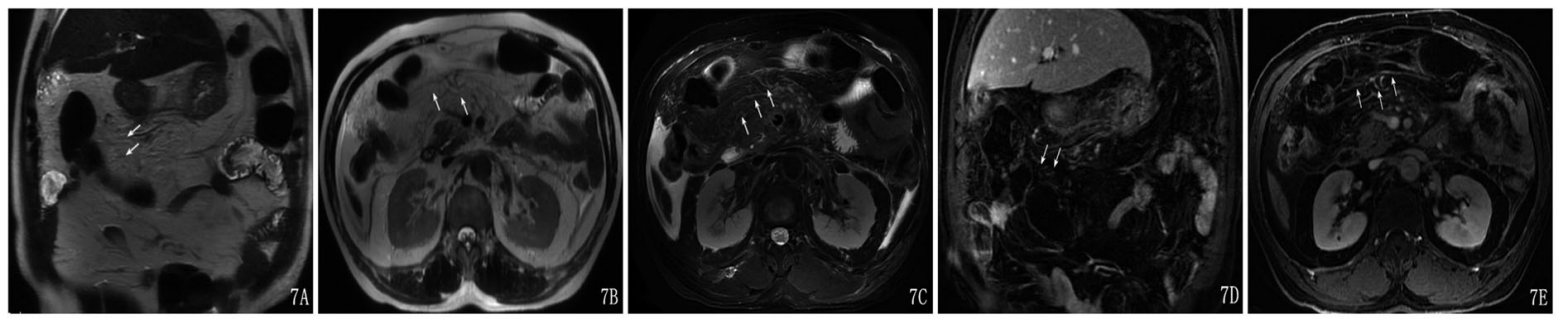
A 46-year-old male with mild AP and TMI was Grade I. Transverse-mesocolon-vessel involvement could be seen on a coronal SSFSE T2-weighted image (A), on an axial SSFSE T2-weighted image (B) and on an axial SSFSE T2-weighted image with fat suppression (C). The transverse mesocolon vessels showed the vessel-lumen expansion and dilated and tortuous lateral-branch small vessels on enhancement of the coronal image (D) and axial image (E).

AP could cause edema, thickening and effusion in the transverse mesocolon. On MRI, the edema and thickening did not show easily on T1WI; it showed as a linear or patchy high signal that was consistent with the running of the mesenteric vessels on T2WI and T2WI+FS (figure 5). The effusions showed as a low signal on T1WI and as an oval or patchy high signal on T2WI and T2WI+FS (figure 6). AP could involve the SMA, SMV and transverse-mesocolon vessels, which showed as blood-vessel-edge unsharpness, vessel-wall thickening, vessel-lumen dilatation and dilated and tortuous lateral-branch small vessels (figure 7). There were 85 cases (40%), 82 cases (39%) and 43 cases (20%) in TMI grades I, II and III, respectively, and 130 patients (61.9%) had transverse-mesocolon-vessel involvement.

In the 76 cases investigated by the dynamic enhanced scan, the mean diameter of the middle colic artery was 1.73±0.36 mm, greater than that of the normal group (1.52±0.23 mm, T = 2.851, P = 0.005). The mean diameter of the vein was 2.44±0.30 mm, greater than that of the normal group (2.19±0.23 mm; T = 3.911, P = 0.000), as was the diameter of the gastrocolic trunk (4.83±0.50 mm vs. 4.36±0.55 mm; T = 3.929, P = 0.000) (Table 1).

**Table 1:** The blood vessels of the transverse mesocolon in normal patients and patients with AP.

	Normal	AP patients	T	P
Middle colic artery	1.52±0.23 mm	1.73±0.36 mm	2.851	0.005
Middle colic vein	2.19±0.23 mm	2.44±0.30 mm	3.911	0.000
Gastrocolic trunk	4.36±0.55 mm	4.83±0.50 mm	3.929	0.000

### 5. The Relationship between Transverse-mesocolon Involvement and MRSI

**Figure 8:**
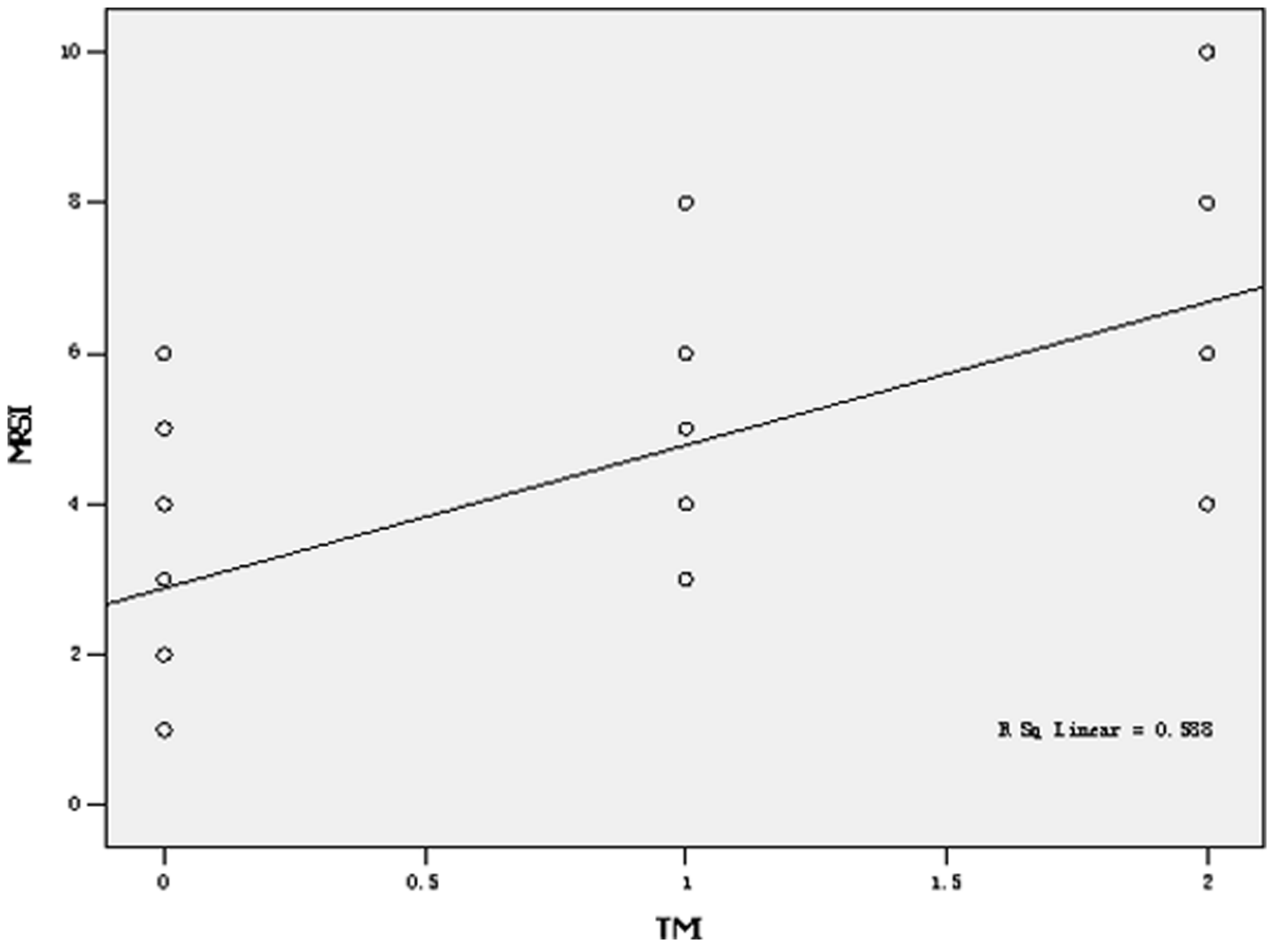
Spearman’s correlation between the MRSI and TMI (r = 0.759, P = 0.000).

Of the 210 patients with AP, 130 (61.9%) showed transverse-mesocolon involvement on the MRI. According to the MRSI, the rates of transverse-mesocolon involvement in mild, moderate and severe AP, respectively, were 25.6% (20/78), 80.9% (93/115) and 100% (17/17) (χ2 = 71.496, P = 0.000). In total, 159 AP patients had SMA and SMV involvement, and 130 patients had transverse-mesocolon-vessel involvement. The rates of transverse-mesocolon-vessel involvement in mild, moderate and severe AP, respectively, were 25.6% (20/78), 80.9% (93/115) and 100% (17/17) (χ2 = 71.496, P = 0.000). From Table 2, we found that with the addition of the MRSI score, the classification of TMI increased. The specific details are listed in Table 2. Among the cases of transverse-mesocolon effusion, three occurred in the left transverse mesocolon, 21 cases occurred in the right, and eight cases occurred on both sides. The TMI on MRI was strongly correlated with the MRSI score (r = 0.759, P = 0.000) (figure 8).

**Table 2:** Transverse-mesocolon involvement and MRSI in patients with AP.

Grade	Mild (n = 78)	Moderate (n = 115)	Severe (n = 17)	P	χ^2^
Grade I	62(79.5%)	23(20%)	0(0%)	0.000	80.842
Grade II	16(20.5%)	64(55.7%)	2(11.8%)	0.000	29.897
Grade III	0(0%)	28(24.3%)	15(88.2%)	0.000	68.314

Chi-squared tests were used to analyze the differences between mild, moderate and severe AP according to MRSI in Grades I, II and III.

### 6. The Relationship between Transverse-mesocolon Involvement and the APACHE-II Score

**Figure 9:**
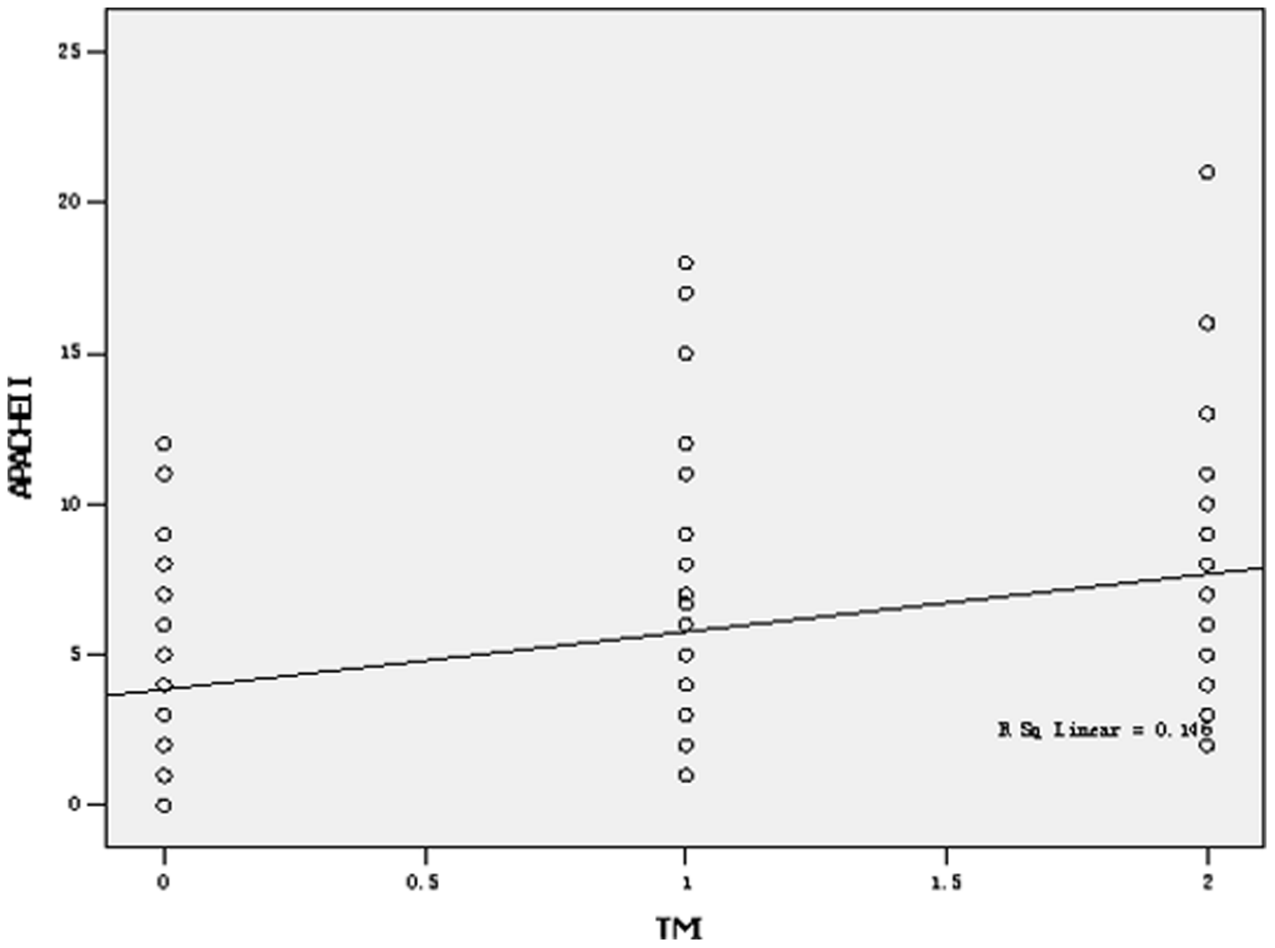
Spearman’s correlation between the APACHE II and TMI (r = 0.384, P = 0.000).

Among the 210 patients with AP, the mean APACHE-II score was 5.11±3.65 points (ranging from zero to 21 points). A total of 163 patients had mild AP (APACHE-II score <8 points), whereas 47 patients had severe AP (APACHE II score ≥8 points). According to the APACHE-II score, the rate of transverse-mesocolon involvement in the mild and severe AP cases, respectively, was 57.7% (94/163) and 76.6% (36/47) (χ^2^ = 5.542, P = 0.019). The rate of transverse-mesocolon-vessel involvement in the mild and severe cases of AP, respectively, was 57.7% (94/163) and 76.6% (36/47) (χ^2^ = 5.542, P = 0.019). From Table 3, we found that with the addition of the APACHE II score, the classification of TMI increased. The specific details are listed in Table 3. Transverse-mesocolon involvement on the MRI was correlated with the APACHE-II score (r = 0.384, P = 0.000) (figure 9).

**Table 3:** Transverse-mesocolon involvement and the APACHE-II scoring system in patients with AP.

Grade	Mild (n = 163)	Severe (n = 47)	P	χ^2^
Grade I	74(45.4%)	11(23.4%)	0.007	7.325
Grade II	66(40.5%)	16(34.0%)	0.425	0.637
Grade III	23(14.1%)	20(42.6%)	0.000	18.124

Chi-squared tests were used to analyze the differences between mild AP and severe AP according to the APACHE-II scoring system in Grades I, II and III.

## Discussion

In this study, the display rates of the middle colic artery, middle colic vein and gastrocolic trunk were 95.0%, 82.5% and 100.0%, respectively. MRI could be used to visualize the transverse mesocolon. All three vessels dilated more in the patients with AP than in the normal group, and the difference was statistically significant. The TMI on the MRI was strongly correlated with the MRSI score (r = 0.759, P = 0.000) and with the APACHE-II score (r = 0.384, P = 0.000). The TMI is a good tool by which to evaluate the severity of AP, reflecting local and systemic complications.

The mesenteries support the organs of the peritoneum, providing a highway for the arteries, veins, nerves and lymphatics [24]. The state of the mesentery artery and vein might signal the need to examine the mesentery [25]. In the normal mesentery, the middle colic artery, middle colic vein and gastrocolic trunk progress naturally and the edge is clear. The gastrocolic trunk delimits the root of the small-bowel mesentery and transverse mesocolon [26]. Imaging of the gastrocolic trunk and the middle colic artery might help determine the position of the transverse mesocolon and discriminate the transverse mesocolon from the gastrocolic ligament and small-bowel mesentery. In the axial images, the middle colic artery took the form of blood vessel lines at the same level as the transverse mesocolon. The right superior colic vein and right gastroepiploic vein were larger branches of the gastrocolic trunk and displayed as long strip vessels in the transverse mesocolon [25]. Our results suggest that axial and coronal images can both be used to visualize the transverse mesocolon on MRI; a combination of the two could provide a more complete picture.

The root of the mesentery is bare and communicates with the anterior pararenal space [27], [28]. AP could involve the mesentery [9], [28], manifesting as the spread of inflammation from the root of the mesentery to the mesentery with involvement of the transverse-mesocolon vessels, transverse-mesocolon edema and effusions [28]. In our study, more transverse mesocolon effusions occurred on the right than on the left, possibly because transverse-mesocolon involvement often started from the roots. Mendez et al. [29] reported that five of the 32 AP patients who received CTs showed mesentery involvement. In our study, 61.9% of the patients showed transverse-mesocolon involvement on the MRI, which is higher than the percentage reported in the literature, suggesting that MR could be used to visualize the transverse-mesocolon involvement. This result might be related to the insensitivity of CT to mild AP (A, B or C grade) [11], [12]. MRI could be used to evaluate inflammatory lesions accurately [13].

The APACHE-II score has the advantage of reflecting systemic complications. An increasing APACHE-II score reflects the general condition of the patient becoming more serious [14], [30]. MRI is a reliable method of grading the severity of acute pancreatitis and has prognostic value [31]. MRSI is better able to predict local complications and prognoses. TMI was strongly correlated with the MRSI and APACHE-II scores and might be used as a supplementary tool to grade the severity of acute pancreatitis.

This study has several limitations. First, the representative transverse-mesocolon vessel showed variation in only a small minority of the patients; our sample size was large, and we judged the position of the transverse mesocolon by examining multiple vessels. Second, transverse-mesocolon involvement might not be caused by pancreatitis. We eliminated some potential confounders when we selected the cases by excluding the patients with diseases that could cause transverse-mesocolon involvement. Transverse-mesocolon involvement was common in the patients with acute pancreatitis and was correlated with the MRSI scores in our study.

MRI could be used to visualize the normal transverse mesocolon and mesocolon involvement in acute pancreatitis. Transverse-mesocolon involvement played an important role in evaluating the severity of AP and might reflect local and systemic complications. TMI might serve as a supplementary indicator of the severity of AP.
